# Optimizing Tracheal Oxygen Tension and Diffusion Ratio When Choosing High-Flow Oxygen Therapy or CPAP for the Treatment of Hypoxemic Respiratory Failure: Insights from Ex Vivo Physiologic Modelling

**DOI:** 10.3390/jcm12082878

**Published:** 2023-04-14

**Authors:** Bill Truschel, Michael I. Polkey

**Affiliations:** 1 Breas Medical AB, SE-435 33 Mölnlycke, Sweden; 2 Royal Brompton Hospital, Guys & St Thomas NHS Foundation Trust, London SE1 4YB, UK

**Keywords:** HFNT, HFOT, hypoxemic respiratory failure, alveolar gas law, Fick’s diffusion, CPAP, oxygen therapy

## Abstract

This article is a review of the physiological and technological processes underpinning high-flow nasal therapy with oxygen (HFNT or HFOT) for the treatment of hypoxemic respiratory failure. A mathematical model was carefully built to represent the relationships between the settings on the HFNT device and the resultant diffusion of oxygen into hypoxemic, arterial blood. The analysis was used to recommend a strategy for setting the flow rate at or above the patient’s peak inspiratory flow when HFNT is used with a blender and equal to the patient’s peak inspiratory rate when bleed-in oxygen is used. The analysis also teaches how to titrate the settings to achieve a desired fraction of inhaled oxygen, ($F_{i}O_{2})$, in the trachea using a simple ratio when bleed-in oxygen is used. The model was used to compare HFNT as a method to improve oxygen diffusion efficacy with other forms of oxygen therapy. The analysis in this article relates the efficacy of HFOT/HFNT to that of CPAP with supplemental oxygen by computing the diffusion ratio of oxygen therapy versus breathing room air. We predicted that in non-atelectatic lungs, when considering oxygenation, HFNT can be equally effective as CPAP with supplemental oxygen therapy for treating hypoxemic respiratory failure.

## 1. Introduction

In a futuristic, dystopian world, when the atmosphere has become saturated with carbon dioxide (CO_2_), a science fiction hero invents the machine to convert CO_2_ to equal parts oxygen (O_2_) and equal parts diamond (C). The wise men of the time harvest the O_2_ and survive, while the foolish masses war over the diamond deposits and perish. Oxygen is vital to our existence because it provides a key ingredient to cellular respiration, and in mammals, the overwhelming physiological method for acquiring O_2_ stores is through breathing.

When the O_2_ transport system from the air to the tissues is impaired, clinicians may employ an O_2_ therapy device with the aim of increasing tissue oxygenation. A necessary preliminary to this is to increase (and optimize) tracheal oxygen tension. The clinician will of course also wish to optimize alveolar oxygenation as well as other downstream factors (for example, gas exchange and cardiac output) that enhance tissue oxygen delivery.

High-flow nasal therapy (HFNT) with supplemental oxygen (HFOT) is minimally obtrusive and thus a therapeutic technique favored by some clinicians and patients. A mathematical model of HFNT machines and a physiologic model of alveolar gas exchange is presented here. This model considers in non-atelectatic lungs the physiological process associated with HFNT and quantifies the effectiveness in terms of oxygenation over a range of device settings and breath patterns, including those that would be difficult or unethical in vivo. This model also provides insight regarding how to best setup HFNT for oxygenation of hypoxemic blood, and it qualifies the effectiveness of HFNT by comparing it to other O_2_ therapy options in terms of oxygen diffusion of blood against the amount of O_2_ used in treatment referenced as a ratio to no treatment.

## 2. Methods

### 2.1. Physiologic Model

The physiologic model for the alveolar gas exchange of oxygen is a combination of Fick’s law and the alveolar gas equation.

#### 2.1.1. Fick’s Law

The transport of dissolved oxygen in air to the cardiovascular system is done at the alveolar epithelium via diffusion through a membrane. The mass transfer of oxygen is done in opposition to the simultaneous, reverse mass transfer of carbon dioxide across the same membrane, with both transfer rates described by Fick’s law of diffusion [1]. The law states that the rate of exchange of oxygen is proportional to a diffusion coefficient, the alveolar surface area, and the differential partial pressure of the oxygen across the membrane. The diffusion coefficient is determined by a multitude of factors, and neglecting instances of altitude sickness, atelectasis, right-left shunting, or surgical lung volume reduction, hypoxemia is a symptom of an abnormally low diffusion coefficient. Diffusion coefficients may be low due to membrane thickening from interstitial disease or fibrosis or other factors that impair gas transfer, such as fluid overload from immunological helpers responding to pathogens or edema. Poor diffusion requires higher oxygen pressure or longer transfer times to achieve normoxia.

The simplified form of Fick’s law is given by (1).

(1) $$\frac{dO_{2}}{dt}=d\cdot A_{sa}\cdot \frac{dP}{T}$$

where

$\frac{dO_{2}}{dt}$ is the transport rate of O_2_;
d is a diffusion coefficient;
$A_{sa}$ is the alveolar surface area;
dP is the difference in tension between the alveolar oxygen and the partial pressure of oxygen in the venous blood of the pulmonary artery (*A*—a gradient);
T is the membrane thickness.

The difference in pressure (dP) may be increased across the alveolar membrane through oxygen therapy (increasing the alveolar oxygen pressure) or through mechanisms such as nitrous oxide (NO) therapy, which reduces the venous pressure through vascular dilation. General lung diffusion can also be improved with the use of diuretics, which both reduce interstitial fluid in the membrane and reduce pulmonary venous pressure through diuresis, patient positioning (proning), or through drugs such as Almitrine Bismesylate to reduce shunting.

In this article, we remain focused on only two factors promoting diffusion specifically related to HFNT and O_2_ therapy devices:

1. The increase in $P_{A}O_{2}$ relative to room air from inhaling pressurized, oxygen-enriched air;
2. An increase or recruitment in alveolar surface area from positive applied pressure (continuous positive airway pressure (CPAP) or positive end expiratory pressure (PEEP)).

The alveolar surface area can be expanded with CPAP or PEEP. In this model, the effect of PEEP on surface area is as follows:

(2) $$A_{sa}=A_{sa\_untreated}\cdot {\left(\frac{FRC+CPAP\cdot C_{lung}}{FRC}\right)}^{2/3}$$

where

$A_{sa\_untreated}$ is the alveolar surface area before CPAP is applied;
FRC is the functional residual lung capacity, and it is assumed that most respiration will occur at this lung volume;
CPAP is the pressure applied;
$C_{lung}$ is the static lung compliance considered to be constant for non-atelectatic or non-distended lungs. Only the lung compliance is considered and assumed be approximately 40 mL/cm H_2_O [2]. The chest wall compliance is not considered when computing the relative increase in alveolar surface area.

#### 2.1.2. Alveolar Gas Equation

The alveolar gas equation describes how to quantify the partial pressure of oxygen in the alveolar gas, $P_{A}O_{2}$ [1].

(3) $$P_{A}O_{2}=F_{i}O_{2}\cdot \left[P_{atm}+P_{app}-P_{H_{2}O}\right]-\frac{P_{a}CO_{2}}{R}$$

where

$F_{i}O_{2}$ is the volume fraction of oxygen in the inhaled gas;
$P_{atm}$ is the atmospheric pressure;
$P_{app}$ is any applied pressure support, PEEP, or CPAP from respiratory equipment;
$P_{H_{2}O}$ is the water vapor pressure in the gas within the alveolus;
$P_{a}CO_{2}$ is the arterial partial pressure of $CO_{2}$;
R is the respiratory quotient (typically 0.8).

The clinical insights obtained from analyzing this Equation (3) are listed in Table 1 with regard to treating hypoxemia via $P_{A}O_{2}$ therapy.

It can be concluded from Table 1 that the primary mechanism associated with oxygen therapy is an increase in $P_{A}O_{2}$ from increasing $F_{i}O_{2}$. Secondary factors are less significant.

### 2.2. Treatment Effectiveness Model Expressed as a Diffusion Ratio

Treatment effectiveness is quantified here as the ratio of the maximum diffusion rate in a treated lung and the maximum diffusion rate in an untreated lung. The diffusion effectiveness of an untreated lung supposes that the patient is not altering his airway structures with pursed-lip breathing or laryngeal narrowing and not voluntarily expanding his alveolar surface area through dynamic inflation of the chest by trapping air. The diffusion effectiveness ratio is expressed as a percentage.

The maximum rate is computed using (1) when the dP is highest. The dP is highest when the enriched $P_{A}O_{2}$ is presented to hypoxemic venous blood in the pulmonary artery. According to (1), the diffusion rate will decrease as the venous return blood is oxygenated; however, for comparative purposes, we only express the maximum diffusion rate as the ratio of the initial rate provided by O_2_ therapy divided by the initial rate of diffusion assuming no therapy (patient breathing room air).

(4) $$Diffusion\;Ratio\%=100\cdot \frac{maximum\;of\;\frac{dO_{2}}{dt}\;treated}{maximum\;of\;\frac{dO_{2}}{dt}untreated}$$

where

$maximum\;of\;\frac{dO_{2}}{dt}\;treated$ occurs when enriched $P_{A}O_{2}$ is presented to hypoxemic $P_{v}O_{2}$ with PEEP;
$maximum\;of\;\frac{dO_{2}}{dt}untreated$ occurs when room air is presented to hypoxemic $P_{v}O_{2}$ without PEEP.

Expressing this effectiveness in this way provides objective comparisons, as settings are adjusted on one device and used to compare HFNT to other types of devices. The effect will always be expressed here as a ratio to the effect of no treatment, and a diffusion ratio of 100% is equivalent to no treatment.

#### 2.2.1. Note Regarding the Clinical Meaning of Diffusion Ratio%

Fick’s law describes diffusion across a membrane with a differential equation where the flux is proportional to the difference in concentration. This article uses Fickian diffusion and reports the clinical efficacy as a value related to the peak diffusion, which is also the initial diffusion rate. The peak rate occurs when the concentration of oxygen in hypoxemic venous blood of the pulmonary artery is at a minimum. Diffusion occurs at a decreasing rate as the concentrations equilibrate. Slower rates require longer transfer time periods to increase the arterial oxygen concentration. It is therefore clinically relevant to consider the peak flux rate during oxygen diffusion as a metric for the overall diffusion efficacy in the context of tidal breathing.

**Figure 1:**
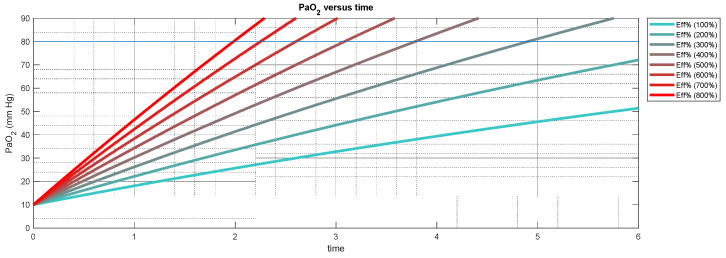
A graphical illustration of the diffusion of oxygen at different efficacy levels compared to untreated patients. The diffusion ratio or maximum rate of diffusion is 800% at $F_{i}O_{2}$ = 1 and 200% at $F_{i}O_{2}$ = 0.3 when compared to breathing room air without treatment. This efficacy refers to the rate at which oxygen is transferred from the alveolus to the venous blood in the pulmonary artery at the initial exposure of the air to the hypoxemic blood across a membrane. The figure illustrates that for higher efficacy, the $P_{a}O_{2}$ will reach normoxia sooner or more likely during tidal breathing during treatment.

Expressed more simply, the efficacy of a treated patient is reported in multiples of that of the untreated patient. (e.g., Diffusion Ratio% may be 300% or three times that of diffusion efficacy under no treatment). The value 300% implies that the maximum diffusion rate of alveolar oxygen into hypoxemic blood during treatment is three times that of the maximum diffusion rate before treatment. This does not indicate that the arterial blood oxygen concentration, $P_{a}O_{2}$, will be three times higher due to treatment. Nor does this value predict that the diffusion capability, such as that measured by the DLCO, or MIGET technique is three times greater when treated. The meaning of this effectiveness measure may be better understood by examining the illustration in Figure 1. In this figure, the efficacy in terms of maximum diffusion rate at an $F_{i}O_{2}$ = 1 is 800% untreated, while at $F_{i}O_{2}$ = 0.3, it is 200% untreated. The enrichment of the blood will occur eight times faster or two times faster when treated and affects both the overall transfer time of alveolar oxygen into venous blood and the arterial oxygen pressure $P_{a}O_{2}$ during spontaneous breathing. With higher diffusion rates, we expect that the treatments are more likely to promote normoxia during tidal breathing.

The model does not predict the $P_{a}O_{2}$ because too many respiratory variables must be considered other than diffusion rate, including breathing rates, diffusion imparities from disease or fluid overload, and the patient’s cardiac output.

#### 2.2.2. Patient Model

The patient model considered is an adult, normocapnic patient with variable breath size and fixed respiratory rate, who lives at the mean elevation of the global population. The assessment of the impact to oxygen diffusion into hypoxemic blood is derived from executing the physiological and machine models with the patient parameters describing this patient. The parameters are listed in Table 2.

**Figure 2:**
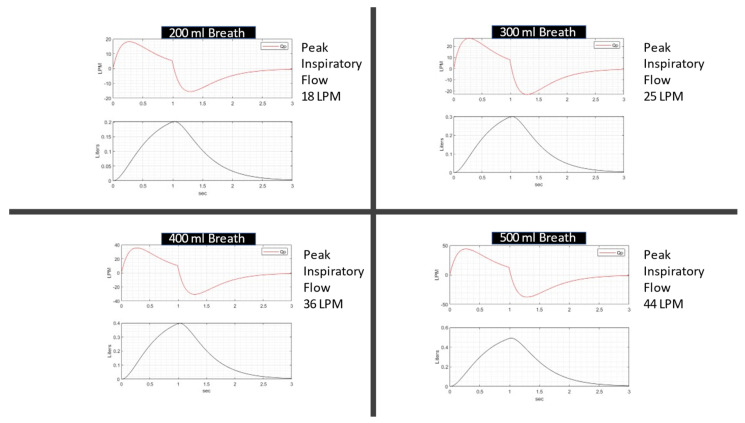
This figure illustrates four differing breath waveforms used throughout this analysis. Flow (Liters/min) is shown in the upper chart of each panel and volume $\left(\mathrm{Liters}\right)$ in the lower chart. The breath sizes range from a shallow 200 mL breath to a normal 500 mL tidal breath. The peak inspiratory flow is listed for each breath. It will become clearer below why the peak inspiratory flow is highly relevant to the clinical effectiveness of the HFNT treatment.

The breath patterns in Figure 2 were generated by adjusting a simulated respiratory drive signal with a single-compartment lung model [8]. The respiratory drive was adjusted to produce a desired tidal volume of 200 mL, 300 mL, 400 mL, and 500 mL to estimate a degree of breath to breath variation on the adult lung model. (R = 10 cm H_2_O/L/s, C = 40 mL/cm H_2_O.)

### 2.3. Modeling the HFNT Device

High-flow nasal therapy with oxygen is a minimally invasive treatment for hypoxemic respiratory failure. In addition to deadspace washout [9] and the secretion clearance benefit provided through breathing humidified high flow [10], HFNT provides benefit to the gas exchange physiology through the following:

(a) Providing supplemental oxygen to increase $F_{i}O_{2}$ and thus increase $P_{A}O_{2}$ [11];
(b) Applying high flow directly to the airway through the nares to provide a small amount of PEEP [12] to expand the gas exchange surface.

The HFNT device is set by the clinician with two parameters:

(1) The cannula flow rate;
(2) The fraction of inhaled oxygen, $F_{i}O_{2}$, in the cannula $F_{i}O_{2}$.

#### 2.3.1. Device $F_{i}O_{2}$ vs. Tracheal $F_{i}O_{2}$

The $F_{i}O_{2}$ measured in the delivery apparatus, nasal cannula, or CPAP tubing (${\mathrm{device}\;F}_{i}O_{2})$ may ignore additional flow sources including leaks or entrained room air. When evaluating patient health using the $P_{a}O_{2}$/$F_{i}O_{2}$ ratio, it is preferred to use the tracheal $F_{i}O_{2}$ rather than device $F_{i}O_{2}$. Tracheal $F_{i}O_{2}$ is measured through a bronchoscopy catheter, expired gas sampling, or through a mathematical model tracking all flow sources including leak and air entrainment [13]. Device $F_{i}O_{2}$ and tracheal $F_{i}O_{2}$ differ more significantly when low flow is delivered, but when using high flow, there may also be differences. This article will solely use the estimated tracheal $F_{i}O_{2}$ when computing diffusion efficiency and ignore the device $F_{i}O_{2}$ that is either set on the machine or measured by the clinician in the patient apparatus.

#### 2.3.2. Estimating Tracheal $F_{i}O_{2}$ for HFNT

In order the estimate the tracheal $F_{i}O_{2}$, it is necessary to consider that the time varying inhalation flow will produce a time-varying tracheal $F_{i}O_{2}\left(t\right)$.

When the inhalation flow is less than the high-flow setting, the $F_{i}O_{2}\left(t\right)$ is equal to the device setting; however, when inhalation flow is greater than the high-flow setting, the patient entrains room air, and the $F_{i}O_{2}$(*t*) is less than the device setting.

**Figure 3:**
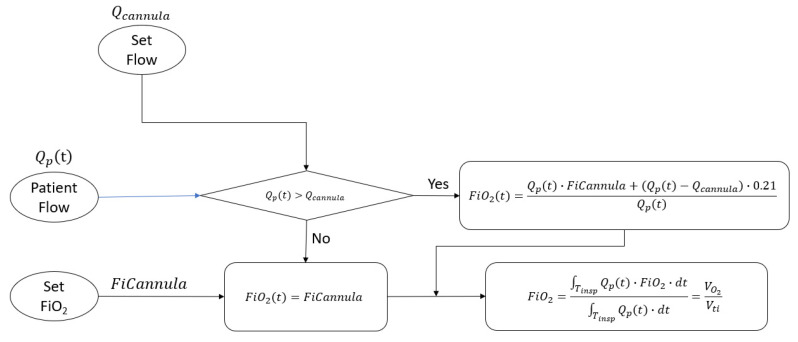
The derivation of tracheal $F_{i}O_{2}$ considering the actual patient flow and the dilution of the oxygen when patient flow may become greater than the cannula flow.

The estimated tracheal volume fraction of $F_{i}O_{2}$ is computed by integrating the time-varying inhaled oxygen flow $F_{i}O_{2}\left(t\right)\cdot Q_{p}\left(t\right)$ divided by the integral of the time-varying inhaled flow $Q_{p}\left(t\right)$. The result is the volume fraction of oxygen inhaled and this is expressed symbolically in Figure 3. It is important to note that this volume fraction is computed once per breath repeatedly, and it cannot be measured directly with a simple oxygen analyzer due to the variability of the concentration over time. To overcome this issue, for example, in Duprez [14], a ventilator set in a controlled ventilation mode held the inspiratory flow constant for the authors to measure $F_{i}O_{2}$.

#### 2.3.3. Heatmap Color Convention for Figures in this Article

The device settings allow two degrees of freedom (a flow setting and an $F_{i}O_{2}$ setting). In presenting the calculated results, namely these two inputs along with the resultant output, a three-dimensional plot is necessary. Here, we present the three-dimensional charts with setting and results as a two-dimensional table with numerical values printed inside a matrix. To prevent retinal fatigue on behalf of the reader, the third dimension associated with the values in the matrix is also presented as a color dimension. In each chart, a numerical value is displayed as a deep red when the value represents the highest clinical benefit to oxygen diffusion. Alternatively, when the numerical value is expressed as a bluer shade, the value represents a lower clinical benefit to the patient’s oxygen diffusion. This convention was chosen to represent the difference in hue between saturated and cyanotic arterial blood. When a fourth dimension is necessary, such as the variability of breath size or breath depth, a three-dimensional surface plot is presented, with the fourth dimension also being represented through color using the same convention described above.

## 3. Results

### 3.1. Tracheal $F_{i}O_{2}$ Estimation during HFNT

The machine model was used with the time-varying breath signals to determine the tracheal $F_{i}O_{2}$ for each setting of flow (4–60 LPM) and $F_{i}O_{2}$ (21–100%). When patient flow was higher than the cannula flow, the model assumed that room air was entrained into the airway by the patient.

**Figure 4:**
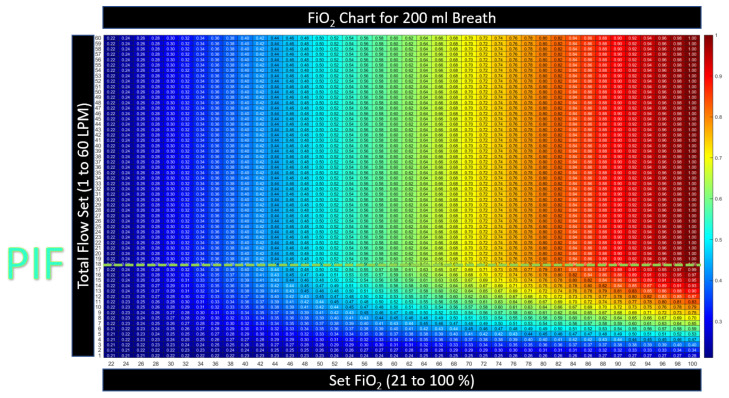
The relationship between the settings and the tracheal $F_{i}O_{2}$ for HFNT on a shallow breath: 200 mL, PIF = 18 LPM. When the flow setting is above the peak inspiratory flow (PIF), the tracheal $F_{i}O_{2}$ is equal to the device $F_{i}O_{2}$, and no change in FiO_2_ is expected in any column above the PIF. The numbers contained within the table are kept small; see the color convention to represent the number, described in Section 2.3.2.

Figure 4 illustrates that the $F_{i}O_{2}$ is maintained when the flow setting is equal to or higher than PIF. The controller automatically increases the oxygen flow with total flow to maintain the device, $F_{i}O_{2}$, and when the cannula flow is sufficient, the model assumes that the patient is not entraining outside air. The model predicts no benefit in tracheal $F_{i}O_{2}$ when the flow is set higher than the PIF. This agrees with in vitro studies by Li [15]. To determine if there is additional expected benefit from higher flow, the complete physiological model that will account for any alveolar surface area expansion relative to the increase in lung volume from the higher flow setting must be examined.

**Figure 5:**
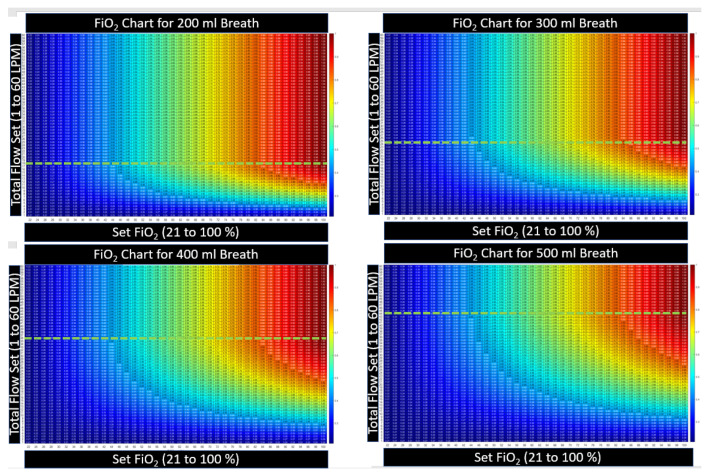
A side-by-side comparison $F_{i}O_{2}$ for the four breath sizes shows that as breath size increases in depth, and PIF increases, the flow setting must be above the maximum breath’s PIF to maintain the set $F_{i}O_{2}$ for all breaths. The green line in each chart above represents the PIF for that size breath. The tracheal $F_{i}O_{2}$ increases below the green line and remains constant in each column above the green line, indicating that setting flow equivalent to PIF is sufficient to achieve the desired $F_{i}O_{2}$. The color convention is described in Section 2.3.2. These charts are meant to show qualitatively, as breaths get larger at the same settings, the charts become more bluish, indicating that tracheal $F_{i}O_{2}$ is lower at the same setting when breath depth increases.

**Figure 6:**
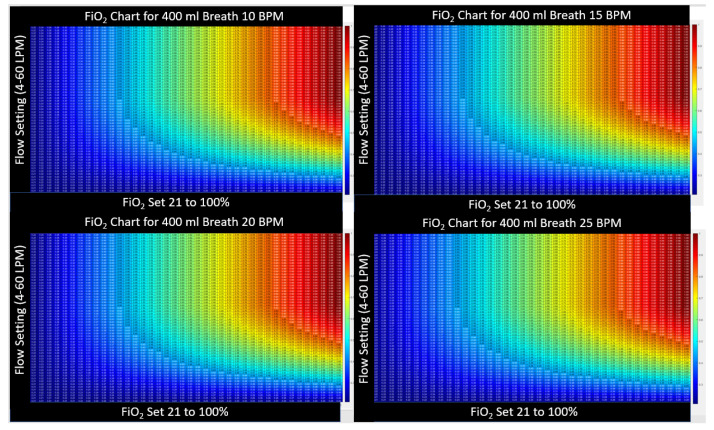
A side-by-side comparison $F_{i}O_{2}$ for a consistent breath size (400 mL, with constant PIF, 36 LPM) at different respiratory rates (10, 15, 20 and 25 breaths per minute (BPM)). The four charts are completely identical, and this teaches that respiratory rate and therefore also minute ventilation does not impact $F_{i}O_{2}$ during treatment with HFNT.

Figure 5 further illustrates that as the peak-inspired flow increases, so must the flow setting to maintain $F_{i}O_{2}$. To insure $F_{i}O_{2}$, the flow setting must be set to the maximum patient inspiratory flow rate accounting for breath-to-breath variability. No other breath parameter, such as respiratory rate, minute ventilation, or I:E ratio plays any role in the HFNT machine to maintain tracheal $F_{i}O_{2}$. As a matter of example, in Figure 6, the breath size is held constant, while the breath frequency (and subsequently patient minute volume) is varied. The tracheal $F_{i}O_{2}$ charts remain identical as the rate changes from 10 BPM to 25 BPM.

### 3.2. Effectiveness of HFNT Treatment vs. No Treatment (Diffusion Ratio)

**Figure 7:**
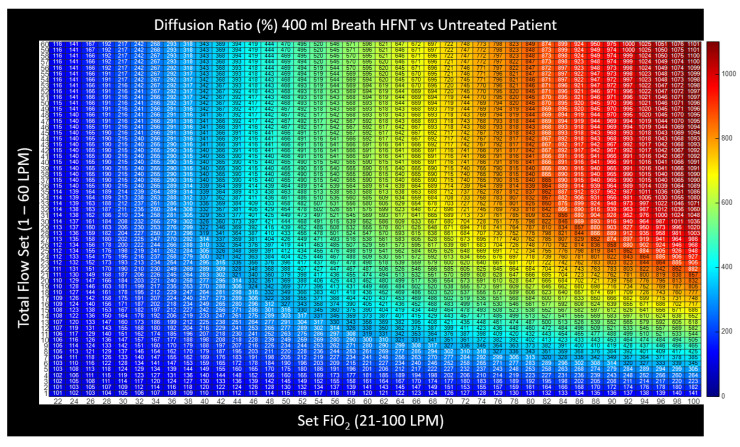
A chart representing the effectiveness of HFNT versus no treatment. The percentage (%) indicates the maximum diffusion rate induced to hypoxemic blood with HFNT treatment at a particular setting as a ratio to the maximum diffusion rate from breathing room air without intervention. This figure’s overall characteristic correlates to the heatmaps for tracheal $F_{i}O_{2}$ in Figure 6, indicating an ANOVA of treatment efficacy is primarily associated with $F_{i}O_{2}$.

**Figure 8:**
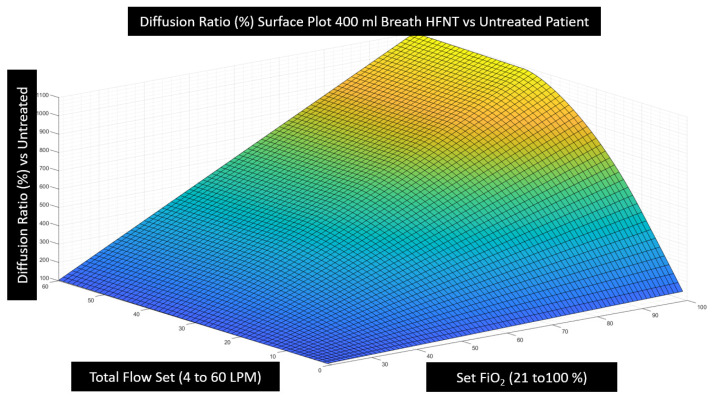
The values in Figure 7 are shown as a surface plot. Effectiveness of the treatment is maximized with increasing the set flow and increasing $F_{i}O_{2}$. If the set flow is below the PIF (36 LPM), we see the diffusion ratio of the treatment is diminished toward no treatment (100%).

**Figure 9:**
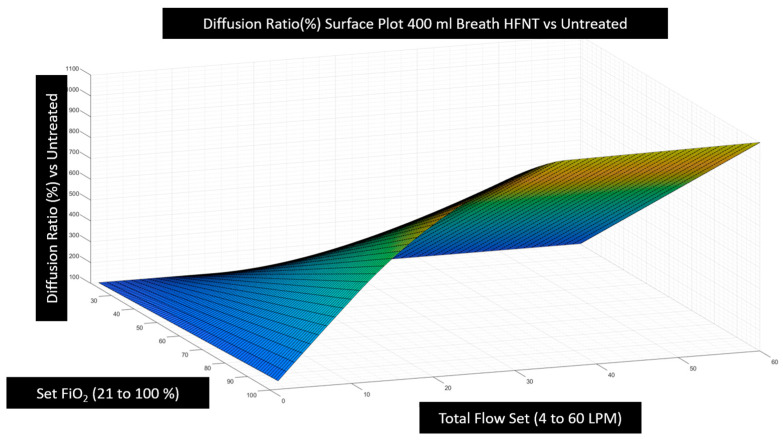
The rotated surface of Figure 8 illustrates how small the diffusion rate benefit is from the small additional PEEP described by Parke et al. [14] due to increasing flow. This is indicated because the surface is flat in the vertical dimension when the flow setting is above the PIF. The diffusion ratio increases as the flow setting is increased to the PIF (36 LPM here), and then, no more benefit at any $F_{i}O_{2}$ setting is incurred from increasing flow above the PIF. This implies that increasing total flow above PIF is simply an unnecessary expenditure of oxygen supplies without perceived clinical benefit to the patient.

**Figure 10:**
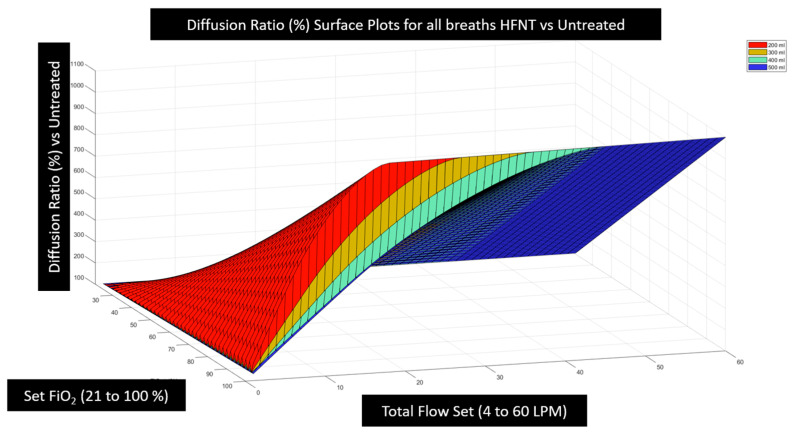
The effectiveness surfaces are drawn together as breath size (PIF) varies. A comparison of HFNT effectiveness at different settings shows that as breaths get larger, the effectiveness of the treatment at the same settings is diminished unless the flow setting is sufficiently large to support the greatest PIF. If the maximum PIF is supported, the effectiveness is maintained; however, there is no additional benefit indicated with increases in flow above the PIF (shown by the flat surface in the vertical dimension on the right side of the plot above). Therefore, the model shows that effectiveness is optimized at a given $F_{i}O_{2}$ setting when the flow is set at or above the PIF.

It was then possible to calculate the diffusion ratio by combining the estimation of tracheal $F_{i}O_{2}$ computed for every combination of breath pattern and device setting (Figure 5) with the alveolar gas Equation (3) and Fick’s law (1) and finally insert these values into Equation (4) to examine the effectiveness of HFNT at various settings as compared with no treatment. Note that diffusion imparities from low hemoglobin, lung disease, or fluid overload, etc., are mathematically cancelled in this analysis when we express the effectiveness as the diffusion ratio because the diffusion coefficient and membrane wall thickness disappear. In expressing effectiveness for a particular patient undergoing treatment versus breathing room air, we have isolated the pure effect of the device and the device settings under examination. This analysis reveals that the overall relationship between diffusion effectiveness correlates closely to the calculation of tracheal $F_{i}O_{2}$, with only small diffusion benefit estimated from other factors such as PEEP induced by HFNT or a small reduction in $P_{a}CO_{2}$ that may occur due to nasopharyngeal washout. In Figure 7, the computational results of HFNT treatment at all settings delivered to a patient with a 400 mL breath is presented as a ratio of the oxygen diffusion rate expected during treatment compared to the oxygen diffusion rate expected without treatment. These same calculations are then expressed in a surface plot in Figure 8, to illustrate the way settings effect diffusion more clearly. In Figure 9, the surface is rotated to illustrate how increasing the total flow setting above the patient’s PIF does not appreciably increase diffusion and therefore is simply an unnecessary expenditure of oxygen supply without benefit to the patient. Finally, in Figure 10, we show that the point of maximum benefit in terms of diffusion of oxygen occurs differently and precisely with matching the total flow setting to the peak inspiratory flow of various breath sizes.

The mathematical model allows for the exploration of multivariate analyses of HFNT effectiveness, including changes in altitude, arterial $CO_{2}$, venous $O_{2},$ humidity, and lung volume, and the results indicate that the primary component of variance in effectiveness is simply a univariate analysis of peak inspiratory flow.

### 3.3. HFNT Setting Recommendations

The results lead to the following recommendations for setting flow and $F_{i}O_{2}$ with the treatment of HFNT.

An algorithm for setting high flow nasal therapy for optimal diffusion efficacy, for greater washout, reduction of work of breathing (WOB) and to provide some level of PEEP *.

**1a. Set the desired** $F_{i}O_{2}$**.**

**2a. Set the Flow ≥ PIF.**

Note: If Flow < PIF, then the tracheal $F_{i}O_{2}$ < Set $F_{i}O_{2}$ and if Flow > PIF, then higher $O_{2}$ flow is required and will deplete $O_{2}$ supply with little clinical benefit to the patient.

* PEEP created from HFNT does not significantly improve diffusion.

### 3.4. Analysis of HFNT without a Blender

When a blender or specialized HFNT device is not available, a clinician may provide HFNT with a turbine and the addition of a bleed-in fixed oxygen flow rate (e.g., with Breas Vivo 3, Mölnlycke, Sweden). The turbine controls the cannula flow, but the $F_{i}O_{2}$ is uncontrolled in these devices.

#### 3.4.1. Tracheal $F_{i}O_{2}$ Model for HFNT without a Blender

**Figure 11:**
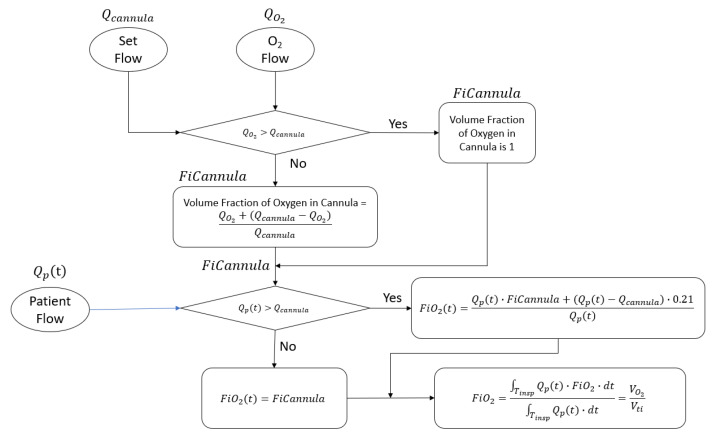
In this figure, the two clinical inputs for HFNT are the set flow and the bleed-in O_2_ flow. The model applies a time-varying patient flow waveform, $Q_{p}\left(t\right)$, and estimates tracheal $F_{i}O_{2}$ based on the mixing of the treatment flow and the entrained room air with the additional dilution occurring when the flow setting is greater than the bleed-in O_2_ flow.

The relationship between tracheal $F_{i}O_{2}$ and the flow setting is derived by the flow chart in Figure 11 and according to the amount of fixed $O_{2}$ flow added to the patient circuit.

**Figure 12:**
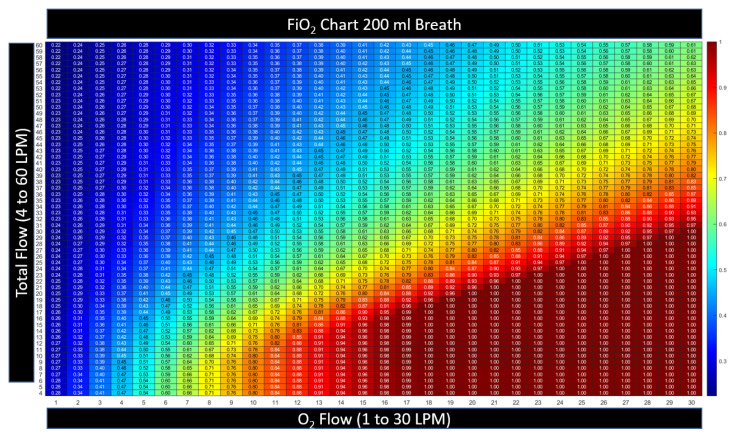
Applying HFNT to a shallow breath with a peak inspiratory flow (PIF) of 18 LPM illustrates clearly how to maximize the tracheal $F_{i}O_{2}$ with settings. $F_{i}O_{2}$ is maximized at each fixed $O_{2}$ flow rate when the total flow is equal to the PIF. Increasing the total flow setting dilutes the device $F_{i}O_{2}$ and therefore the tracheal $F_{i}O_{2}$.

Figure 12 reveals that the relationship between tracheal $F_{i}O_{2}$ and settings is “upside down” when compared to the relationship derived from traditional HFNT settings (compare to Figure 3). This is because dilution occurs when the flow setting is high and the $O_{2}$ flow rate is fixed. In traditional HFNT with a blender, the $O_{2}$ flow rate is increased in proportion to the flow rate automatically by the machine.

**Figure 13:**
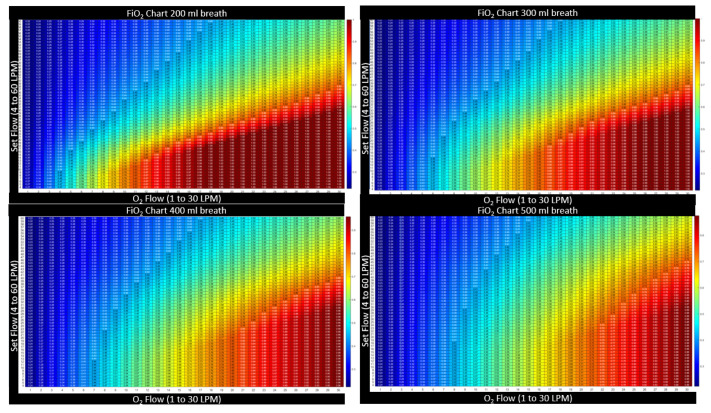
An illustration of all four $F_{i}O_{2}$ charts for the four candidate breaths shows how both total flow settings and $O_{2}$ flow rate must increase with breath size. The deeper breaths with higher PIF require higher settings to achieve the desired tracheal $F_{i}O_{2}$. Here also note, indicated by the color trends on the charts, that increasing the flow setting always dilutes the tracheal $F_{i}O_{2}$.

Figure 13 illustrates how to control the tracheal $F_{i}O_{2}$ with flow settings and bleed-in oxygen rates for these devices without a blender. The tracheal $F_{i}O_{2}$ can reach levels of 1.0 when the total flow is equal to the PIF, and the $O_{2}$ flow is greater than or equal to the total flow settings, but there exists a complex relationship between breath pattern, flow setting, and bleed-in flow rate otherwise. In all cases the tracheal $F_{i}O_{2}$ become more bluish as the flow setting is increased at a fixed $O_{2}$ flow. This indicates, that when using a turbine without a blender for HFNT, as flow settings are increased, the $O_{2}$ is always diluted.

**Figure 14:**
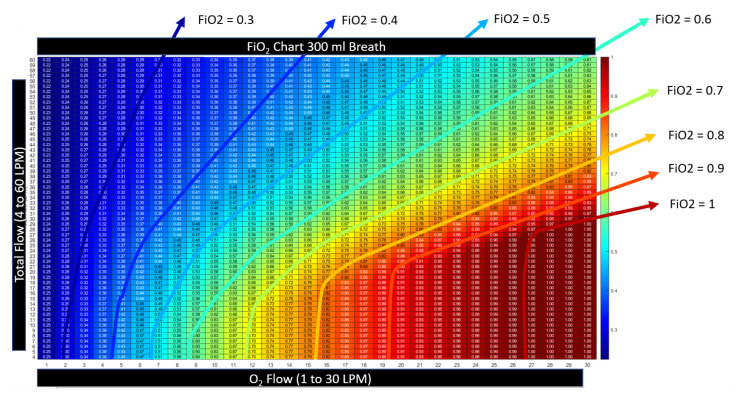
The complex relationship between settings and $F_{i}O_{2}$ values is shown by this chart for HFNT with bleed-in oxygen. For this 300 mL breath, the ability to maintain an $F_{i}O_{2}$ is achieved with increasing total flow and $O_{2}$ flow bleed-in rate together. The PIF for the 300 mL breath is 25 LPM after the total flow equal or exceeds PIF, $F_{i}O_{2}$, and the relationship can be determined by a ratio of the device settings. When flow is less than PIF, the $F_{i}O_{2}$ calculation requires knowledge of the breath pattern.

Figure 14 shows graphically, the complex relationship between the flow setting, the amount of $O_{2}$ flow added into the circuit and the tracheal $F_{i}O_{2}$ for a single size (300 mL) breath. The simplest way to control the tracheal $F_{i}O_{2}$ is explained below in an algorithm below in Section 3.4.2.

#### 3.4.2. Guidelines for Setting HFNT with Bleed-In Oxygen

The results lead to the following recommendations for setting flow and $F_{i}O_{2}$ with the treatment of HFNT. Here, it is necessary to set the flow equal to the PIF, and any breath variability will result in a reduction of $F_{i}O_{2}$.

The method for setting tracheal $F_{i}O_{2}$ when using HFNT with bleed in oxygen is as follows:

1a. Increase the Flow setting to match PIF *.

**2a. Provide a bleed-in** $O_{2}$ **rate by**

$$O_{2}\;Flow=Flow*\;\frac{desired\;F_{i}O_{2}-0.21}{0.79}$$

* Formula in (2a) does not apply if the flow setting is less than PIF because the patient entrains room air during the breath and does not apply if the flow setting is greater than PIF because the machine dilutes the bleed-in oxygen.

If Flow is not equal to PIF the following relationship is true.

**2b.** $\;tracheal\;F_{i}O_{2}<\frac{0.79O_{2}\;Flow+0.21\;Flow}{Flow}$

It may be necessary in these devices to choose a slightly higher $F_{i}O_{2}$ when the patient is breathing more irregularly due to the relationship between PIF and $F_{i}O_{2}$ that always lowers $F_{i}O_{2}$ when PIF and the flow setting are unequal.

### 3.5. Comparison of HFNT to CPAP with Oxygen

To provide perspective of these effects and determine how effective HFNT is for treating hypoxemic respiratory failure compared to other forms of oxygen therapy, the model was used to compare HFNT therapy to that of CPAP plus bleed-in oxygen therapy in non-atelectatic lungs. When applying CPAP, the clinician has the option to entrain oxygen at or near the patient interface or in a port at the rear of the machine. Because of the intentional leak in the CPAP circuit, $F_{i}O_{2}$ is greater when the option of entraining oxygen at the mask is chosen. However, CPAP manufactures will recommend the use of the rear oxygen connection port because it allows the machine to better monitor the patient’s spirometry. Both techniques ($O_{2}$ entrained at the mask and $O_{2}$ connected to the back of the machine) were modeled, and the expected diffusion effectiveness was calculated using the physiological model in Section 2.2.

**Figure 15:**
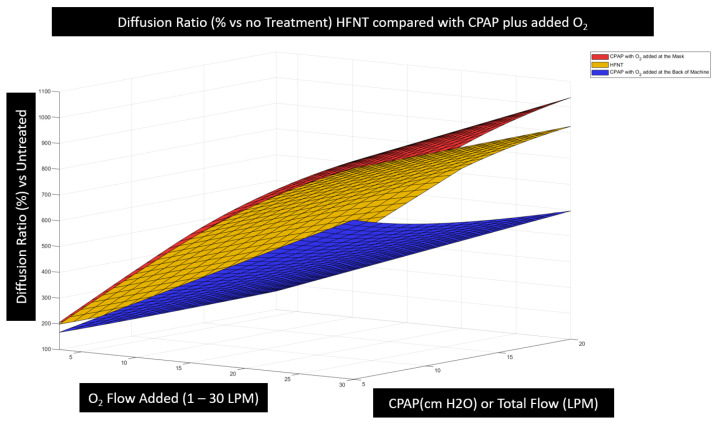
This figure illustrates a comparison of the diffusion efficacy with variable settings for HFNT^3^, CPAP plus $O_{2}$ entrained at the mask, and $O_{2}$ added to the back of the CPAP. The diffusion efficiency describes the maximum diffusion rate of $O_{2}$ in hypoxemic arterial blood for each device/settings combination compared to no treatment. The comparison shown here uses the breath size of 500 mL and plots in one dimension the flow of oxygen used in the therapy. The flow of oxygen is not constant in HFNT; however, it was computed as an average flow used by the HFNT blender during the breath. In the second dimension, the CPAP pressure or the flow setting from HFNT is plotted on the same scale, reminding the reader that the PEEP and total flow are correlated in HFNT. This third dimension indicates effectiveness according to equation (4). The red and yellow surfaces describing the effectiveness of the CPAP with $O_{2}$ entrained at the mask (red) and HFNT (yellow) are similar, while CPAP is below a nominal 15 cm H_2_0. The blue surface describes the least-effective method: CPAP with $O_{2}$ entrained at the device.

Figure 15 illustrates that with respect to $O_{2}$ flow rate, the HFNT machine performs as well as the CPAP machine with $O_{2}$ entrained at the mask. The only differences in effectiveness arise when the higher CPAP pressures > 15 cm H_2_O begin to expand the alveolar surface area and contribute to higher $P_{A}O_{2}$. It is also apparent that the HFNT is superior in efficacy at the same $O_{2}$ flow rate when compared with CPAP machines when $O_{2}$ is added at the device.

^3^HFNT with and without a blender has the same efficacy vs. $O_{2}$ consumption despite the differences in setup because the delivery method is the same.

## 4. Discussion

Ex vivo modeling demonstrates that in non-atelectatic lungs, HFNT is as effective for promoting oxygen diffusion as CPAP when oxygen is entrained at the mask and is superior to CPAP where oxygen is entrained at the back of the device. Specifically, the benefit from alveolar expansion from CPAP pressure or increased end expiratory lung volume (EELV) provides no significant benefit to oxygenation in non-atelectatic lungs.

### 4.1. Significance of the Findings

Our model suggests that the magnitude of PIF is sufficient for optimizing the flow setting. It was reported previously by Lie et al. [14] that setting flows 10, 20, or 30 LPM above the PIF may provide some clinical benefit to hypoxemic patients; however, a close examination of the data presented by Lie shows that with regard to $F_{i}O_{2}$, we are in agreement with Lie that there is no benefit to increasing flow above PIF. This was confirmed in vitro by Lie who discovered there is no clinical benefit to increasing flow above PIF through an examination of the $S_{p}O_{2}$ measurements that are also maximized when flow is equal to but not greater than PIF. We speculate that the benefit reported by Lie for higher flow (in the $S_{p}O_{2}$/$F_{i}O_{2}$ ratio and the ROX index measurements) may have been imputed by the authors due to a poor polynomial fitting of the $F_{i}O_{2}$ prediction (see Figure 3a in Lie et al.). Moreover, the recommendation of higher-than-PIF flow settings may also result in worsening patient tolerance, as reported by Basile [16].

The mathematical model for tracheal $F_{i}O_{2}$ here is also in agreement with the bench model of Duprez et al. [14]; however, the limitation of their bench model does not provide the precise relationship between tracheal $F_{i}O_{2}$ and settings because the flow pattern was limited to a constant flow in volume control ventilation, and the use of an $O_{2}$ analyzer can only measure instantaneous $O_{2}$ concentration and not the true $F_{i}O_{2}$. Here, the $F_{i}O_{2}$ is represented accurately and carefully as a volume fraction of inhaled oxygen, and this volume fraction is influenced by the breath pattern when inspiratory flow is not constant, as it will vary during spontaneously breathing patients treated by HFNT.

The recommended settings for HFNT hypoxemic respiratory failure are simple; setting the flow equal to the PIF of the patient allows for precise control of the tracheal $F_{i}O_{2}$.

When HFNT is used with bleed-in oxygen, the flow should also be set near or equal to the PIF because when flow is less than PIF, the patient will entrain air, and the tracheal $F_{i}O_{2}$ will become unpredictable but always less than the delivered $F_{i}O_{2}$. Conversely, when the flow is greater than PIF, the $F_{i}O_{2}$ is diluted, and treatment is less effective at treating hypoxemia. In practice, this relationship can be used to practice a technique where flow is increased while observing the patient’s $S_{p}O_{2}$. Increasing the flow further than the optimal point will dilute the treatment, and we would expect the $S_{p}O_{2}$ to fall.

Peak inspiratory flow will vary according to disease and disease state; clinicians using HFNT to some extent must thus make an assessment based on both factors. The most extreme condition of reduced PIF occurs in patients with neuromuscular disease and inspiratory muscle weakness, but low PIF has also been reported in chronic obstructive disease [17], where the apposition of the diaphragm is such that force generation is not effectively transmitted to the thoracic cavity [18]. Any condition that increases neural drive will tend to increase peak inspiratory flow, including but not limited to hypoxia and infection. In fact, in most conditions where HFNT is required, there will be a remarkably high neural drive. It is also worth observing that if the patient has previous lung function tests, the flow volume loop may provide an accurate guide to PIF.

### 4.2. Critique of the Method

The analysis here is limited by the lack of closed-loop control of the patient’s chemoreceptive system. It can be expected that as oxygen is administered to patients who are both responsive and unresponsive to the treatment, the respiratory drive and concentration of venous oxygen pressure will change with time. It is therefore important to observe and update settings, particularly when the depth of breathing or need for higher $F_{i}O_{2}$ may be changing over time such that the HFNT settings should be updated concurrently.

It was of interest that the change in lung volume modelled with CPAP was modest at the CPAP pressures conventionally used (see Figure 15); however, this assumes a “healthy” lung. Several disease-specific factors may modify the relationship between the CPAP pressure applied (low or negligible in the case of HFNT) and lung volume. First, in patients at the lower end of the pressure volume curve (typically those with obesity or neuromuscular weakness), CPAP may push the patient into the steeper part of the pressure volume (PV) curve, thus reducing the work of breathing and lessening the likelihood of failure of the respiratory muscle pump, which would be manifest as hypercapnia. In patients with COPD, the effect of increased CPAP is hard to predict; too little may move the patient to a flatter part of the PV curve, increasing work of breathing; on the other hand, a CPAP pressure that matches the intrinsic positive expiratory pressure (PEEPi) should overcome flow limitation and move the patient to a steeper part of the curve. The clinical picture, of course, is further compounded where there is asymmetric disease; this could be left/right (e.g., in the case of a lobar pneumonia), upper zone vs. lower zone (e.g., in the case of restriction due to ascites or abdominal splinting), anterior vs. posterior (as may occur in acute lung injury), or completely random as a function of the disease, for example, as in the case of a large bulla. In all these examples, there will be regional variation of lung/circulatory interaction. However, we submit that these considerations should not preclude the clinician trying to optimize tracheal $F_{i}O_{2}$ with matched settings on the device to the patient as part of the overall care plan.

## 5. Conclusions

In non-atelectatic lungs, HFNT is equivalent to CPAP with oxygen entrained at the mask and superior to oxygen entrained at the rear of the machine. A simple guide for junior clinicians would be to target the flow setting to the peak inspiratory flow rate mandated by the patient’s disease and clinical condition.

## Figures and Tables

**Table 1:** Factors Effecting Alveolar Oxygen Pressure.

Parameter	Correlation to Oxygen Diffusion	Impact	Treatment Method
$F_{i}O_{2}$	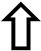	Significant, $F_{i}O_{2}$ application can increase $P_{A}O_{2}$ by a factor of 5	$O_{2}$ therapy
$P_{atm}$	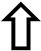	Small impact to $P_{A}O_{2}$ in most inhabitable areas, but low atmospheric pressure can lead to altitude sickness	hyperbaric chamber or moving the patient to sea level
$P_{app}$	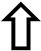	Small impact to $P_{A}O_{2}$ as applied pressures are insignificant in relation to atmospheric pressure; however, the applied pressure also aids diffusion by increasing surface area.	CPAP, PEEP, or MV
$P_{H_{2}O}$	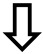	$\mathrm{Humidity}\;\mathrm{lowers}\;P_{A}O_{2}$, but is necessary especially with HFNT to prevent injury and dehydration.	Active or Passive heated humidification, natural humidification through mucosal membranes or HME
$P_{a}CO_{2}$	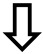	$\mathrm{Poor}\;\mathrm{ventilation}\;\mathrm{slightly}\;\mathrm{reduces}\;P_{A}O_{2}$	Inspiratory Pressure Support or Expiratory Pressure therapy to aid obstructive disease
R	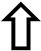	Does not vary far from typical values of 0.8	Dietary changes

**Table 2:** The parameter values used in this article.

Parameter	Symbol	Value Used	Reference
Functional Residual Capacity	FRC	3 L	[3]
Alveolar SA untreated	$A_{SA\_untreated}$	118 m^2^	[4]
Atmospheric Pressure	$P_{atm}$	99 kPa ^1^	[5]
Alveolar Water Vapor Pressure	$P_{H_{2}O}$	47.08 mm Hg	[6]
Arterial CO_2_ Pressure	$P_{a}CO_{2}$	46 mm Hg	[1]
Respiratory Quotient	R	0.8	[1]
Venous O_2_ Pressure in the Pulmonary Artery during Hypoxemia	$P_{v}O_{2}$	32 mm Hg	[7]

^1^ Atmospheric Pressure is calculated from a 194 m elevation and using the relationship $P_{atm}=101325{\left(1-0.0000225577*Elevation\right)}^{5.2588}$.
